# Pulmonary Rehabilitation Accelerates the Recovery of Pulmonary Function in Patients With COVID-19

**DOI:** 10.3389/fcvm.2021.691609

**Published:** 2021-07-20

**Authors:** Pengfei Zhu, Zhengchao Wang, Xiaomi Guo, Zhiyong Feng, Chaochao Chen, Ai Zheng, Haotian Gu, Yu Cai

**Affiliations:** ^1^Department of Cardiology, Wuhan Fourth Hospital, Puai Hospital, Tongji Medical College, Huazhong University of Science and Technology, Wuhan, China; ^2^Tongji Hospital, Tongji Medical College, Huazhong University of Science and Technology, Wuhan, China; ^3^Department of Ultrasound, Wuhan Asia General Hospital, Wuhan, China; ^4^Department of Rehabilitation, Wuhan Fourth Hospital, Puai Hospital, Tongji Medical College, Huazhong University of Science and Technology, Wuhan, China; ^5^British Heart Foundation Centre of Research Excellence, King's College London, London, United Kingdom

**Keywords:** pulmonary training, corona virus disease 2019, pulmonary function, pulmonary rehabilitation, 2019-nCoV

## Abstract

**Objectives:** To evaluate the effect of in-hospital pulmonary rehabilitation (PR) on short-term pulmonary functional recovery in patients with COVID-19.

**Methods:** Patients with COVID-19 (*n* = 123) were divided into two groups (PR group or Control group) according to recipient of pulmonary rehabilitation. Six-min walk distance (6MW), heart rate (HR), forced vital capacity (FVC), forced expiratory volume in 1 s (FEV_1_), diffusing capacity of the lung for carbon monoxide (DL_CO_), and CT scanning were measured at the time of discharge, 1, 4, 12, and 24 weeks.

**Results:** At week one, both PR group and Control group showed no significant changes in pulmonary function. At 4 and 12 weeks, 6MW, HR, FVC, FEV_1_, and DL_CO_ improved significantly in both groups. However, the improvement in the PR group was greater than the Control group. Pulmonary function in the PR group returned to normal at 4 weeks [FVC (% predicted, PR vs. Control): 86.27 ± 9.14 vs. 78.87 ± 7.55; FEV1 (% predicted, PR vs. Control) 88.76 ± 6.22 vs. 78.96 ± 6.91; DLCO (% predicted, PR vs. Control): 87.27 ± 6.20 vs. 77.78 ± 5.85] compared to 12 weeks in the control group [FVC (% predicted, PR vs. Control): 90.61 ± 6.05 vs. 89.96 ± 4.05; FEV1 (% predicted, PR vs. Control) 94.06 ± 0.43 vs. 93.85 ± 5.61; DLCO (% predicted, PR vs. Control): 91.99 ± 8.73 vs. 88.57 ± 5.37]. Residual lesions on CT disappeared at week 4 in 49 patients in PR group and in 28 patients in control group (*p* = 0.0004).

**Conclusion:** Pulmonary rehabilitation could accelerate the recovery of pulmonary function in patients with COVID-19.

## Introduction

Corona Virus Disease (COVID-19) caused by a novel coronavirus named as Severe Acute Respiratory Syndrome (SARS)-CoV (Corona Virus)-2 has been rapidly occurring the world and is not completely controlled till now (1, 2). Transmissions through fecal-oral route and ocular are also considered to be possible while evidences are not sufficient till now (3, 4). All age groups are susceptible to SARS-CoV-2, while the elderlies and people with underlying diseases are more likely to develop severe conditions such as severe pneumonia and respiratory failure in a short period of time (2). The therapeutic principles of COVID-19 include general treatment (vital sign monitoring, mechanical ventilation, etc.), drug therapy (anti-infection drugs, traditional Chinese medicine, etc.), pulmonary rehabilitation (PR), nutrition management and mental support.

Pulmonary rehabilitation, as a comprehensive intervention including exercise training, education and behavioral changes that aims to improve the physical and psychological condition in patients with respiratory disease and to promote high long-term quality of life. It has also been confirmed to be an important part of the integrated care strategy for chronic obstructive pulmonary disease (COPD) (5, 6). Its positive effects in preoperative pulmonary rehabilitation were also discovered including reducing the sensation of dyspnea, reducing muscle strength loss associated with dyspnea, and improving psychologic states (7). As for infectious disease of respiratory system, Hsieh et al. (8) found that survivors of acute respiratory distress syndrome (ARDS) caused by influenza A (H1N1) who received pulmonary rehabilitation for 2 months had improved pulmonary function, exercise capacity, and quality of life.

Therefore, the aim of present study was to evaluate the effect of in-hospital pulmonary rehabilitation on short-term pulmonary functional recovery in patients with COVID-19.

## Methods

### Patients and Data Collection

We conducted a perspective observational study in patients with COVID-19.

Participants were recruited from Puai Hospital, Wuhan Forth Hospital and Huazhong University of Science and Technology, and were divided into two groups according to whether patients received in-hospital pulmonary rehabilitation. Patients who underwent in-hospital pulmonary rehabilitation were based on the clinical judgements by attending physicians. No patients were directly involved in the design, planning and conception of this study. Inclusion criteria were: (1) patients with COVID-19; (2) able to receive pulmonary rehabilitation; (3) no co-infection of other pathogene; (4) sign the informed consent. Exclusion criteria include: (1) suffering from high blood pressure, diabetes, or other chronic or basic diseases; (2) COVID-19 recurrence during the follow-up period. (3) infection of other pathogene during the follow-up period. (4) pregnancy before or during the follow-up period. Data were collected at the time of discharge and 1, 4, 12, 24 weeks after discharge. The study was approved by Chinese Clinical Trial Registry (ChiCTR2000031751).

### Pulmonary Rehabilitation

In the PR group, all patients underwent a standardized rehabilitation scheme (ref) when their clinical condition was stable and capable of PR. Detailed PR protocol as follow: (1) allow patients to maintain regular movement, such as chest expansion and ambulation, in the isolation ward for at least 1 h per day while monitoring heart rate and respiratory rate during movement to avoid overexertion in terms of heart and lung function; (2) provide respiratory control training: Help the patients sit in an upright position to avoid orthopnea. If the patients could not sit upright, lift the head of bed by 60 degrees. Let the patients relax their shoulder muscles by placing one hand on the chest and the other on the abdomen, instruct the patients to deeply breathe in through their nose and breathe out through their mouth to expand the lower chest. (3) pursed lip breathing: Keep the same patient position as with respiratory control. Let the patients breathe in through their nose, hold their breath for 2 s, then deeply breathe out using their abdomen for 3–5 s with their mouth pursed as if they are whistling; this increases the expiratory resistance and prolongs the expiratory time. For (2) and (3) above, the patients were trained repeatedly for 10–15 min each and 4 times per day. The patients could train along with light music if possible. If any discomfort occurred, the training should be stopped immediately.

### Outcome Measures

Six-min walk distance (6MW), Heart rate (HR), forced vital capacity (FVC), forced expiratory volume in 1 s (FEV_1_), and Diffusing capacity of the lung for carbon monoxide (DL_CO_) were measured. CT scanning was conducted at discharge, 4, and 24 weeks. FVC and FEV_1_ were measure using spirometry. Spirometry was performed using the Medical Graphics CPXD (Minneapolis, MN, US). Diffusing capacity of the lung for carbon monoxide (DL_CO_) were assessed using the rebreathe technique and a mass spectrometer (Perkin Elmer, St. Louis, MO, USA) as previously described (9, 10). CT scan was conducted using a 64-slice spiral CT machine (NeuSoft, NeuViz64). The CT images was evaluated by two experienced imaging clinicians. If their opinions were different, a third clinician was invited to make the final decision.

### Statistical Analysis

Statistical analysis was completed using SPSS 21.0. Baseline differences between groups were analyzed by Student's *t*-test for continuous data and by the χ^2^ test for categorical data. Continuous data are expressed as the means ± SDs, and the normality of distribution was tested by a QQ plot. The data were analyzed using Student's *t*-test and repeated measures analysis of variance (ANOVA). As for repeated measures ANOVA, *post-hoc* test of *p*-value was adopted by Bonferroni correction and effect size was expressed as eta-square. A value of *p* < 0.05 was considered statistically significant. Because of a small sample size, *p*-valued between 0.05 and 0.1 was marked with specific value.

## Results

**Figure 1:**
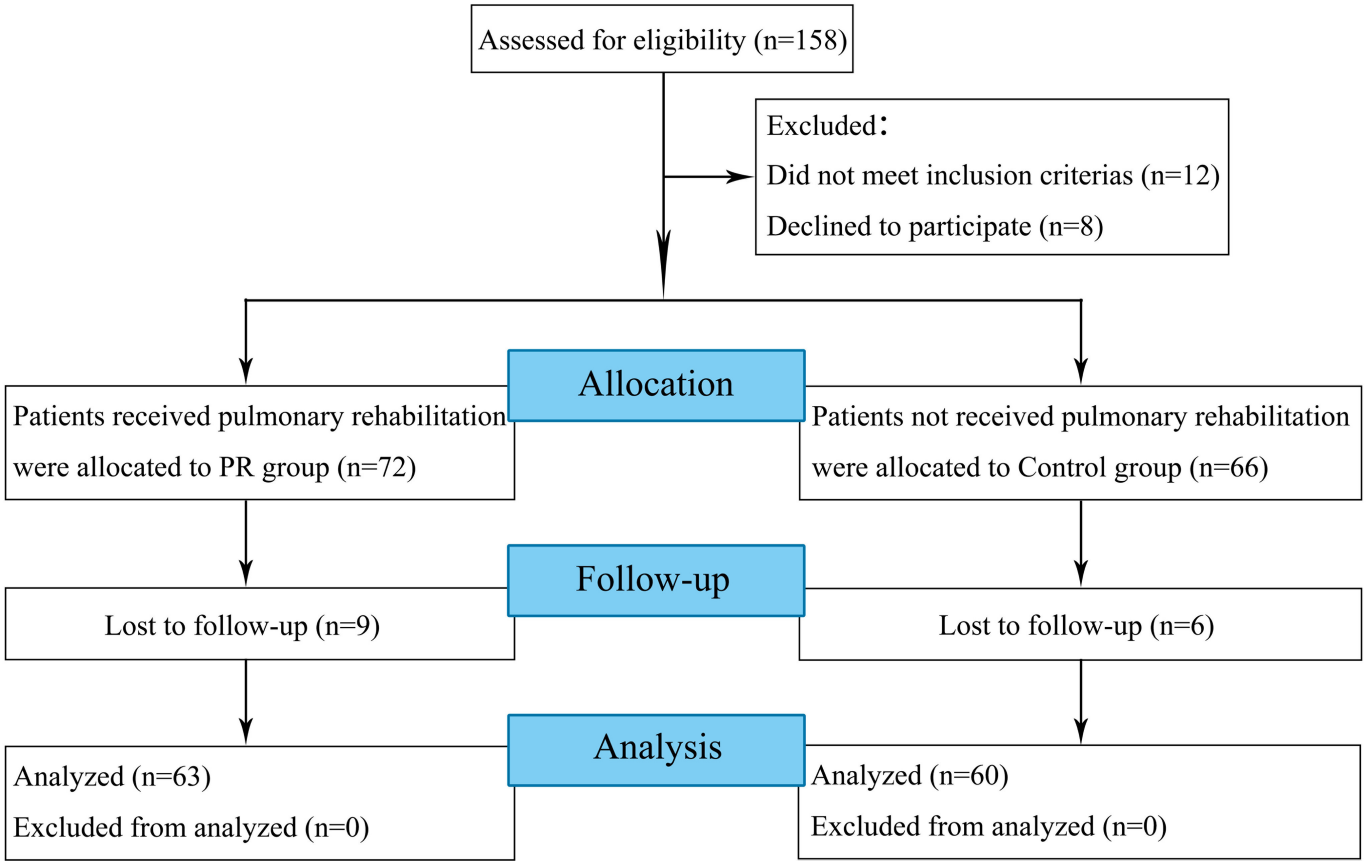
Flow diagram of the study design.

A total of 158 participants were screened between February 1st 2020 to March 31st 2020, out of whom 20 patients were excluded because of not meeting the inclusion criteria or declined to participate in this study (Figure 1). Fifteen participants were lost to follow-up before 4 weeks follow-up. Baseline demographics were shown in Tables 1–3.

**Table 1:** General characteristics in PR and control groups.

	**No. (%)**		***p*-value**
	**PR Groupbreak (*n* = 63)**	**Control Groupbreak (*n* = 60)**	
Age (years)	36.59 ± 7.01	35.47 ± 7.58	0.40
Gender			0.53
Male	34	29	
Female	29	31	
Blood pressure (mmHg, at discharge)			
Systolic pressure	116.3 ± 4.4	115.8 ± 5.2	0.57
Diastolic pressure	78.7 ± 3.2	78.1 ± 3.5	0.32
Weight (kg, at discharge)	62.4 ± 11.3	64.0 ± 10.9	0.43
Height (cm, at discharge)	167.6 ± 13.2	167.8 ± 12.9	0.93
Personal habits			
Smoking	7 (11.1)	9 (15.0)	0.52
Drinking	4 (6.3)	3 (5.0)	0.75
Education			0.66
Junior high school or below	12	9	
High school or vocational school	19	24	
College degree	16	17	
Bachelor degree	12	8	
Postgraduate degree or above	4	2	

*PR, pulmonary rehabilitation*.

**Table 2:** Clinical characteristics in PR and control groups.

	**No. (%)**		***p*-value**
	**PR Groupbreak (*n* = 63)**	**Control Groupbreak (*n* = 60)**	
**Clinical Presentation**			
Fever	62 (98.8)	59 (98.3)	1.00
Dry cough	45 (71.4)	41 (68.3)	0.71
Headache	5 (7.9)	4(6.7)	1.00
Sore throat	7 (11.1)	5 (8.3)	0.40
Myalgia	21(33.3)	18 (30.0)	0.69
Fatigue	24 (38.1)	19 (31.7)	0.46
Dyspnoea	28 (44.4)	21 (35.0)	0.29
Rhinorrhoea	13 (20.6)	11 (18.3)	0.75
Nausea & vomiting	18 (28.6)	19 (31.7)	0.71
Diarrhea	12 (19.0)	10 (16.7)	0.73
Length of Hospital stay (days)	21.18 ± 4.98	21.94 ± 3.24	0.32

*PR, pulmonary rehabilitation*.

**Table 3:** Results of laboratory examination at discharge.

	**PR Groupbreak (*n* = 63)**	**Control Groupbreak (*n* = 60)**	***p*-value**
**Blood Count**			
WBC (×10^9^/L)	7.14 ± 3.41	6.86 ± 2.99	0.63
Lymphocyte count (×10^9^/L)	0.62 ± 0.08	0.66 ± 0.09	0.20
PLT at discharge (×10^9^/L)	243 ± 99	216 ± 71	0.09
Hemoglobin (g/dL)	118 ± 23	125 ± 17	0.10
**Coagulation Function**			
PT (s)	14.1 ± 3.3	13.2 ± 1.4	0.08
APTT (s)	37.6 ± 9.0	37.2 ± 6.2	0.82
D-dimer (mg/L)	1.7 ± 2.4	1.3 ± 1.9	0.24
**Blood Biochemistry**			
TP (g/L)	64.5 ± 10.3	66.5 ± 7.2	0.21
Albumin (g/L)	34.6 ± 5.8	37.5 ± 6.4	0.10
ALT (U/L)	35 ± 19	40 ± 22	0.14
AST (U/L)	30 ± 15	34 ± 19	0.20
TB (μmol/L)	11.8 ± 5.5	12.5 ± 6.2	0.51
Sodium (mmol/L)	137.7 ± 5.3	138.5 ± 3.3	0.30
Potassium (mmol/L)	4.1 ± 0.5	3.9 ± 0.4	0.09
Creatinine (μmol/L)	71.2 ± 27.5	69.1 ± 20.4	0.62
BUN (mmol/L)	5.2 ± 2.1	5.4 ± 2.3	0.66
LDH (U/L)	239 ± 133	213 ± 127	0.27
CK-MB (U/L)	10.9 ± 8.5	11.8 ± 7.7	0.53
**Infection-Related Biomarkers**			
CRP (mg/L)	23 ± 34	18 ± 25	0.34
PCT (ng/ml)	0.18 ± 0.45	0.11 ± 0.18	0.21

*PR, pulmonary rehabilitation; RBC, red blood cell, WBC, white blood cell; PT, prothrombin time; PLT, platelet; APTT, activated partial thromboplastin time; FBG, fasting blood glucose; TP, total protein; ALT, alanine transaminase; AST, aspertate aminotransfera; TB, total bilirubin; BUN, blood urea nitrogen; LDH, lactate dehydrogenase; CK-MB, creatine kinase–MB; CRP, hypersensitive C-reactive protein; PCT, procalcitonin*.

6MW and HR were shown in Table 4. At the time of discharge, 6MW distance in PR group was longer than the Control group and the HR was lower than the Control group, but did not reach significant. At the time of week 1 and 4, there were significant improvements of 6MW and HR in PR group compared to those at the time of discharge (week 1: 495.88 ± 34.67 vs. 470.83 ± 35.70 *p* < 0.05 and 83.24 ± 8.46 vs. 97.05 ± 14.24 *p* < 0.001; week 4: 557.94 ± 38.44 vs. 514.22 ± 43.47 *p* < 0.01 and 78.59 ± 6.73 vs. 88.61 ± 9.37 *p* < 0.001). However, in the Control group, only an improvement of 6MW was found at 4 weeks and was smaller than the PR group. At 12 and 24 weeks, 6MW and HR were similar in two groups.

**Table 4:** Six-min walk distance and heart rate.

	**Discharge**	**1 week**	**4 weeks**	**12 weeks**	**24 weeks**
**6MW (m)**					
PR group	462.12 ± 31.61	495.88 ± 34.67 ^*^	557.94 ± 38.44 ^*^^†^	584.41 ± 20.12 ^*^^†^	598.71 ± 22.35 ^*^^†^^‡^
Control group	448.56 ± 31.10	470.83 ± 35.70	514.22 ± 43.47 ^*^^†^	573.11 ± 29.20^*^^†^^‡^	590.33 ± 19.88 ^*^ ^†^^‡^
PR vs. control	*p* > 0.05	*p* < 0.05	*p* < 0.01	*p* > 0.05	*p* > 0.05
p and η^2^ for ANOVA	*p*_time_ < 0.001, $\eta_{\text{time}}^{2}$ = 0.932, p_group_ < 0.05, $\eta_{\text{group}}^{2}$ = 0.124, *p*_time*group_ < 0.001, $\eta_{\text{time}*group}^{2}$ = 0.168				
**HR (beats/min)**					
PR group	90.71 ± 9.30	83.24 ± 8.46 ^*^	78.59 ± 6.73 ^*^	76.06 ± 6.09 ^*^^†^	76.06 ± 6.09 ^*^^†^
Control group	97.44 ± 10.39	97.05 ± 14.24	88.61 ± 9.37	78.61 ± 9.37 ^*^^†^^‡^	77.00 ± 6.16 ^*^^†^^‡^
PR vs. control	*p* = 0.052	*p* < 0.001	*p* < 0.001	*p* > 0.05	*p* > 0.05
p and η^2^ for ANOVA	*p*_time_ < 0.001, $\eta_{\text{time}}^{2}=$ 0.778, *p*_group_ < 0.05, $\eta_{\text{group}}^{2}$ = 0.143, *p*_time*group_ < 0.001, $\eta_{\text{time}*group}^{2}$ = 0.332				

*PR, pulmonary rehabilitation; 6MW, 6-min walk distance; HR, heart rate*.

*P-value of PR. vs. Control was from Student's t-test between two groups*.

*P-value of the comparison between different times was from post-hoc test with Bonferroni correction for multiple comparisons [C5(2)]:*

* *p <0.05/10 vs. discharge*;

† *p < 0.05/10 vs. 1 week*;

‡ *p < 0.05/10 vs. 4 weeks*.

The measurements of FVC and FEV_1_ is shown in Table 5. At the time of discharge and week 1, FEV_1_ in the PR group was significantly larger than that in the Control group. There was no significant difference in FVC between the two groups at week 1 (2.05 ± 0.26 vs. 1.91 ± 0.21, *p* = 0.096). Although FVC and FEV1 improved significantly in both groups, there was greater improvement in the PR groups than the Control group at week 4. FEV_1_ and FVC in the PR group exceeded 80% of predicted values at 4 weeks [FVC (% predicted): 86.27 ± 9.14 vs. 78.87 ± 7.55, *p* < 0.05; FEV1 (% predicted) 88.76 ± 6.22 vs. 78.96 ± 6.91, *p* < 0.001]. At 12 and 24 weeks, there were no significant difference in FEV_1_ and FVC between two groups and FEV1 and FVC reached 90% of predicted values. There was no significant change in FEV_1_ to FVC ratio during the entire follow-up period.

**Table 5:** Forced vital capacity and forced expiratory volume in 1 s.

	**Discharge**	**1 week**	**4 weeks**	**12 weeks**	**24 weeks**
**FVC (L)**					
PR group	2.05 ± 0.26	2.11 ± 0.29	2.75 ± 0.30 ^*^^†^	2.89 ± 0.22 ^*^^†^	2.95 ± 0.15 ^*^^†^
Control group	1.91 ± 0.21	2.02 ± 0.19	2.51 ± 0.20 ^*^^†^	2.86 ± 0.12 ^*^^†^^‡^	2.91 ± 0.10 ^*^^†^^‡^
PR vs. control	*p* = 0.096	*p* > 0.05	*p* < 0.05	*p* > 0.05	*p* > 0.05
p and η^2^ for ANOVA	*p*_time_ < 0.001, $\eta_{\text{time}}^{2}$ = 0.947, *p*_group_ = 0.053, $\eta_{\text{group}}^{2}$ = 0.109, *p*_time*group_ < 0.05, $\eta_{\text{time}*group}^{2}$ = 0.288				
**FVC (% predicted)**					
PR group	64.25 ± 7.94	66.29 ± 9.14	86.27 ± 9.14 ^*^^†^	90.61 ± 6.05 ^*^^†^	92.64 ± 3.27 ^*^^†^
Control group	60.04 ± 6.28	63.46 ± 6.32	78.87 ± 7.55 ^*^^†^	89.96 ± 4.05 ^*^^†^^‡^	91.51 ± 2.62 ^*^^†^^‡^
PR vs. control	*p* = 0.090	*p* > 0.05	*p* < 0.05	*p* > 0.05	*p* > 0.05
p and η^2^ for ANOVA	*p*_time_ < 0.001, $\eta_{\text{time}}^{2}$ = 0.946, *p*_group_ = 0.061, $\eta_{\text{group}}^{2}$ = 0.102, *p*_time*group_ < 0.05, $\eta_{\text{time}*group}^{2}$ = 0.295				
**FEV**_**1**_ **(L)**					
PR group	1.52 ± 0.12	1.54 ± 0.14	2.12 ± 0.11 ^*^^†^	2.25 ± 0.10 ^*^^†^^‡^	2.29 ± 0.14 ^*^^†^^‡^
Control group	1.43 ± 0.11	1.48 ± 0.09	1.88 ± 0.12 ^*^^†^	2.24 ± 0.10 ^*^^†^^‡^	2.31 ± 0.13 ^*^^†^^‡^
PR vs. control	*p* < 0.05	*p* < 0.05	*p* < 0.001	*p* > 0.05	*p* > 0.05
p and η^2^ for ANOVA	*p*_time_ < 0.001, $\eta_{\text{time}}^{2}$ = 0.980, *p*_group_ < 0.01, $\eta_{\text{group}}^{2}$ = 0.302, *p*_time*group_ < 0.001, $\eta_{\text{time}*group}^{2}$ = 0.499				
**FEV**_**1**_ **(% predicted)**					
PR group	63.62 ± 5.82	64.54 ± 7.11	88.76 ± 6.22 ^*^^†^	94.06 ± 0.43 ^*^^†^	95.83 ± 5.29 ^*^^†^^‡^
Control group	59.81 ± 4.94	61.58 ± 5.29	78.96 ± 6.91 ^*^^†^	93.85 ± 5.61 ^*^^†^^‡^	97.01 ± 5.79 ^*^^†^^‡^
PR vs. control	*p* < 0.05	*p* < 0.05	*p* < 0.001	*p* > 0.05	*p* > 0.05
p and η^2^ for ANOVA	*p*_time_ <0.001, $\eta_{\text{time}}^{2}$ = 0.938, *p*_group_ <0.05, $\eta_{\text{group}}^{2}$ = 0.152, *p*_time*group_ <0.001, $\eta_{\text{time}*group}^{2}$ = 0.198				
**FEV**_**1**_**/FVC (%)**					
PR group	74.73 ± 5.89	73.67 ± 8.08	77.70 ± 6.70	78.15 ± 5.96	77.62 ± 4.25
Control group	74.99 ± 5.55	74.39 ± 6.63	75.32 ± 5.43	78.30 ± 4.37	78.55 ± 5.35
PR vs. control	*p* > 0.05	*p* > 0.05	*p* > 0.05	*p* > 0.05	*p* > 0.05
p and η^2^ for ANOVA	*p*_time_ <0.001, $\eta_{\text{time}}^{2}$ = 0.565, *p*_group_ > 0.05, $\eta_{\text{group}}^{2}$ = 0.004, *p*_time*group_ > 0.05, $\eta_{\text{time}*group}^{2}$ = 0.210				

*PR, pulmonary rehabilitation; FVC, forced vital capacity; FEV1, forced expiratory volume in 1s*.

*P-value of PR. vs. Control was from Student's t-test between two groups*.

*P-value of the comparison between different times was from post-hoc test with Bonferroni correction for multiple comparisons [C5(2)]:*

* *p < 0.05/10 vs. discharge*;

† *p < 0.05/10 vs. 1 week*;

‡ *p < 0.05/10 vs. 4 weeks*.

DL_CO_ was shown in Table 6. At the first week after discharge, no improvements were discovered in DL_CO_. Meanwhile, the DL_CO_ of PR group was higher than Control group (19.65 ± 2.12 vs. 17.03 ± 1.94, *p* < 0.01). Significant improvements were discovered at 4 weeks, while level of DL_CO_ in the PR group was higher than the Control group [DLCO (% predicted): 87.27 ± 6.20 vs. 77.78 ± 5.85, *p* < 0.001]. At 12 and 24 weeks, DL_CO_ reached normal level and had no significantly differences between two groups.

**Table 6:** Diffusing capacity of the lung for carbon monoxide.

	**Discharge**	**1 week**	**4 weeks**	**12 weeks**	**24 weeks**
**DL**_**CO**_ **[ml/(min·mmHg)]**					
PR group	18.53 ± 2.03	19.65 ± 2.12	21.76 ± 2.19 ^*^	22.88 ± 2.12 ^*^^†^	22.94 ± 2.33 ^*^^†^
Control group	16.00 ± 1.46	17.03 ± 1.94 ^*^	18.83 ± 1.86 ^*^	21.50 ± 2.38 ^*^^†^^‡^	22.72 ± 2.16 ^*^^†^^‡^
PR vs. control	*p* < 0.001	*p* < 0.01	*p* < 0.001	*p* = 0.079	*p* > 0.05
p and η^2^ for ANOVA	*p*_time_ <0.001, $\eta_{\text{time}}^{2}$ = 0.753, *p*_group_ <0.01, $\eta_{\text{group}}^{2}$ = 0.271, *p*_time*group_ <0.01, $\eta_{\text{time}*group}^{2}$ = 0.145				
**DL**_**CO**_ **(% predicted)**					
PR group	74.36 ± 6.59	78.81 ± 6.57	87.27 ± 6.20 ^*^^†^	91.99 ± 8.73 ^*^^†^	92.12 ± 8.32 ^*^^†^
Control group	66.24 ± 6.20	70.32 ± 7.46	77.78 ± 5.85 ^*^^†^	88.57 ± 5.037 ^*^^†^^‡^	93.94 ± 8.29 ^*^^†^^‡^
PR vs. control	*p* < 0.01	*p* < 0.01	*p* < 0.001	*p* > 0.05	*p* > 0.05
p and η^2^ for ANOVA	*p*_time_ <0.001, $\eta_{\text{time}}^{2}$ = 0.740, *p*_group_ <0.01, $\eta_{\text{group}}^{2}$ = 0.283, *p*_time*group_ <0.01, $\eta_{\text{time}*group}^{2}$ = 0.143				

*PR, pulmonary rehabilitation; FVC, forced vital capacity; DL_CO_, diffusing capacity of the lung for carbon monoxide*.

*P-value of PR. vs. Control was from Student's t-test between two groups*.

*P-value of the comparison between different times was from post-hoc test with Bonferroni correction for multiple comparisons [C5(2)]:*

* *p < 0.05/10 vs. discharge*;

† *p < 0.05/10 vs. 1 week*;

‡ *p < 0.05/10 vs. 4 weeks*.

**Figure 2:**
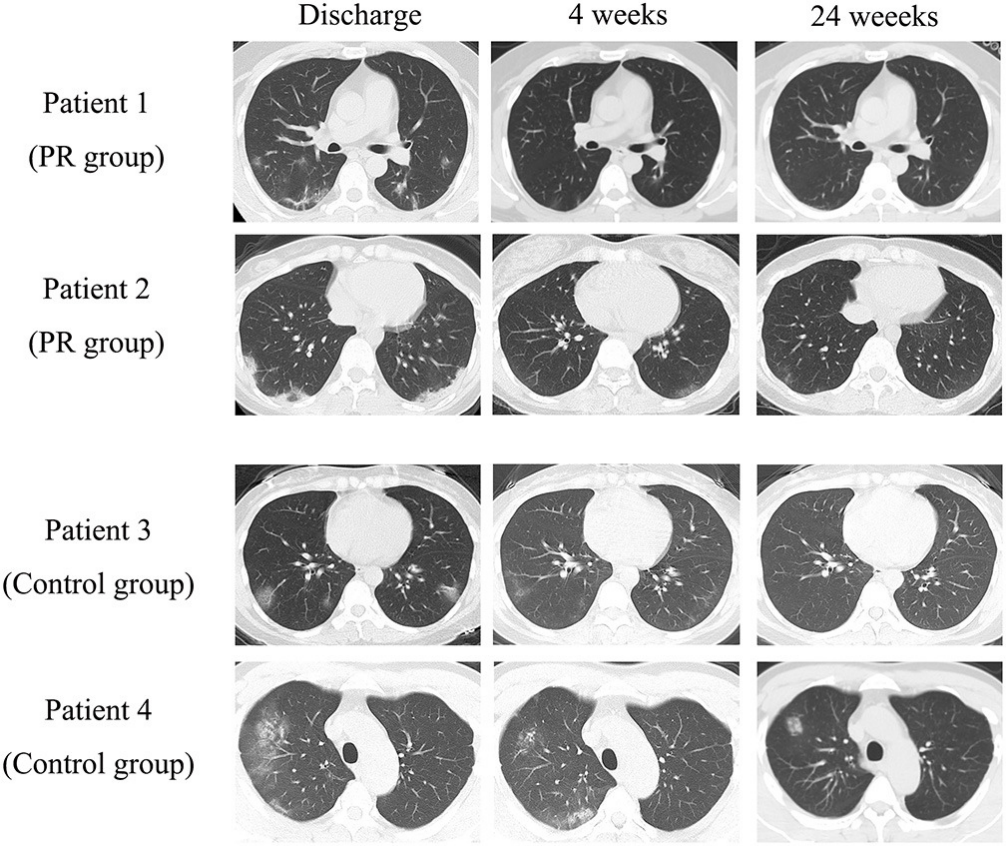
Typical CT imaging of each group.

As shown in Figure 2, in the PR group, little parenchymal bands with group-glass opacity were observed at the time of discharge in all patients. The lesions of 49 patients (77.8%) in PR group basically disappeared at 4 weeks follow-up and no changes were discovered at 24 weeks. The CT images of 60 patients (95.2%) in PR group were basically normal at 24 weeks. In the control group, little parenchymal bands with more group-glass opacities were observed at the time of discharge in all patients. At 4 weeks follow-up, some group-glass opacities still existed in CT images of 32 patients (53.3%). The lesions of only 28 patients (46.7%, *p* = 0.0004 vs. PR group) in control group basically disappeared at 4 weeks follow-up and no changes were discovered at 24 weeks. The CT images of 56 patients (93.3%, *p* = 0.65 vs. PR group) in control group were basically normal at 24 weeks.

## Discussion

The main physiological change in patient recovery from COVID-19 is poorer cardio-pulmonary function, and lower FVC, FEV_1_, and DL_CO_. Meanwhile most of the values of FEV_1_/FVC were still abnormal. The main imaging changes from CT scanning were little parenchymal bands with residual group-glass opacity. As a result, the pathologic changes in the lung of patients after discharge might be: (1) residual unabsorbed exudative lesion; (2) mild lung fibrosis. These changes result in the functional disorders include: (1) decreasing in lung capacity; (2) decreasing in lung compliance; (3) decreasing in diffusion function. However, all impairments disappeared within 12 weeks, which means the pathological and functional changes are reversible.

The residual lesions of lung function are not rarely in viral pneumonia. Studies have discovered that survivors from SARS had significantly impaired pulmonary function, limited physical and psychology function, and reduced life quality (11, 12). Regarding influenza A virus H1N1, a study found that over half of these patients had signs of more severe abnormal pulmonary function, including diffusion disorders and small airway dysfunction, 1 year after discharge (13). From our results, we found that the residual lesions of lung function caused by SARS-CoV-2 is relatively short-term and reversible. It might attribute to the relatively lower virulence of the virus or the participants we included were not severe and critical.

Pulmonary rehabilitation is a comprehensive intervention that includes but is not limited to exercise training, education and behavioral changes with the aim to improve the physical and psychological conditions of people with respiratory disease and promote long-term quality of life (6). Previous studies have confirmed the positive effects of pulmonary rehabilitation on pulmonary diseases such as COPD and H1N1 pneumonia (8, 12, 14). Besides, pulmonary rehabilitation has been proved to benefit the lung function and life quality in interstitial lung diseases such as idiopathic pulmonary fibrosis and interstitial pneumonias (15–17). Based on clinical practice, the program of pulmonary mainly contained three aspects: (1) physical training, (2) respiratory training, and (3) psychological regulation. Therefore, there are three main benefits of PR: (1) improve the patients' exercise capacity, (2) improve the patients' pulmonary function, and (3) improve the patients' psychological state. During the whole follow-up from the time of discharge to 24 weeks later, we can find that the pulmonary function of PR group was basically normal at 4 weeks, while Control group was basically normal at 12 weeks. As a result, the pulmonary rehabilitation could accelerate the recovery of pulmonary lesions and cardio-pulmonary function. According to the changes in CT imaging, we suspected that the effects of pulmonary rehabilitation may attribute to the promotion in absorption of exudation and fibrosis lesions, result in improvement of lung capacity, compliance, and diffusion function.

Because of the flexibility, feasibility and low cost, pulmonary rehabilitation could be a relatively practical way to improve patient condition. Most of patients suffered from COVID-19 are mild and common type, which makes it easy to carry out pulmonary rehabilitation. As for critical patient, whether, when, and how to carry out pulmonary rehabilitation should be further considered. Moreover, the intensity of training relies on the patients' condition; hence, the therapists should pay more attention each patient's vital signs and subjective feelings to not only maximize the effectiveness of the training but also avoid adverse events.

The main limitation of our study is that we only reported the results of 24 weeks follow-up, whether COVID-19 have sequela in respiratory system or other systems should be further studied. On the other hand, the characteristics of socio-economic of patients might affect patients' choice for accepting pulmonary rehabilitation, which might also lead to a better recovery. However, the socio-economic data were not available, which could be another limitation for this research.

In conclusion, pulmonary rehabilitation could accelerate the recovery of pulmonary function for COVID-19 patients.

## Data Availability Statement

The raw data supporting the conclusions of this article will be made available by the authors, without undue reservation.

## Ethics Statement

The studies involving human participants were reviewed and approved by Wuhan Fourth Hospital. The patients/participants provided their written informed consent to participate in this study.

## Author Contributions

PZ, HG, and YC conceived and designed the study. YC and PZ contributed to the literature search. CC, ZF, and AZ contributed to data collection. AZ, ZF, and XG contributed to data analysis. PZ and XG contributed to data interpretation. ZW contributed to the figures. ZW, HG, and PZ drafted the article. All authors contributed to the article and approved the submitted version.

## Conflict of Interest

The authors declare that the research was conducted in the absence of any commercial or financial relationships that could be construed as a potential conflict of interest.
